# Book Reviews

**Published:** 1805-11-01

**Authors:** 


### CRITICAL ANALYSIS
### OF THE
### RECENT PUBLICATIONS
### ON THE
### DIFFERENT BRANCHES OF PHYSIC, SURGERY, AND MEDICAL PHILOSOPHY.

*Cases of Pulmonary Consumption, &c. treated with Uva-Ursi, to which are added some practical Observations.* By ROBERT BOURNE, M. D. Alarichian Professor of the Practice of Physic in the University of Oxford, Fellow of the London College of Physicians, One of the Physicians to the Radcliffe Infirmary, and late Fellow of Worcester College. 8vo. pp. 293. Oxford.

NOTWITHSTANDING the extensive improvement in the modern science and practice of Medicine, the frequency and fatality of pulmonary consumption is still a subject of too just and general observation. A considerable interest therefore must necessarily be excited by every prospect, however distant and obscure, of adding to the number of resources which art supplies to combat this gigantic and destructive malady.

The work now before us is prefaced with an apology for the delay of its publication. When the treatise was far advanced in the press, the author received a communication from a physician of eminence, stating, that the result of his experiments on the antiphthisical properties of uva ursi were by no means favourable to the supposition of its efficacy. In consequence of this communication, Dr. Bourne resolved to withhold his intended publication, until a more enlarged experience had established the validity of his previous conclusions, and the treatise has at length appeared, containing six additional cases, which tend to confirm the author in his his former opinions concerning the efficacy of uva-ursi in pulmonary consumption.

This plant has long been a medicine of reputed utility in certain affections of the urinary organs. It was in consequence of its apparent virtue in counteracting a protracted and obstinate disorder in the urinary passages, attended with emaciation, and all the characteristics of hectic fever, that our author was induced to make trial of its efficacy in phthisis pulmonalis, and other affections rendered in some measure analogous to genuine pulmonary consumption by the decided existence of hectic irritation.

After a recital of the case above alluded to, Dr. Bourne, in the work under consideration, minutely details the symptoms and method of treatment in sixteen separate cases, which are arranged under four general heads. The first eight are supposed by our author to be instances of "true pulmonary consumption in its first stage," the ninth, tenth, and eleventh, of this disorder in a confirmed state, attended with purulent expectoration; the two succeeding were affections of the lungs attended with expectoration of pus, but which, nevertheless, were not genuine phthisis; and the three last were "cases of hectic, in which the lungs appeared not to be primarily affected, or not at all."

In the majority, however, of the above cases, the uva-ursi was not had recourse to without auxiliary combinations, and in some instances its employment was for a time entirely suspended.

In the cases which are recited in the appendix, the medicine in question appears generally to have received a fairer trial, and to have been attended with a more decided effect; and the following may perhaps be regarded, both with respect to the characters of the disorder and the course pursued in its treatment, as containing evidence at least equal with any recorded in the work of the anti-phthisical powers of uva-ursi.

"T. A. of Oxford, a married man, twenty-six years of age, called upon me for advice on December 10, 1804. He was a pressman at the Clarendon Printing House; and it accidentally occurred that a sheet of my publication caught his eye, and, from the nature of the subject, attracted his attention in its way through the press. The sheet was the one in which the ninth case was contained, which case appearing to him in all leading points, very similar to his own, he became desirous of submitting his complaints to me; hoping I might direct the same medicine as had been employed on that occasion.

He had had a cough eleven weeks. The cough had troubled him by day chiefly; but it troubled him a little by night also. It had been attended with considerable expectoration. From the commencement of the cough, he had felt an uneasiness on the right side of the chest, which had obliged him to lie on the left side; he could not go to sleep when lying either on his right side or on his back. His breath at the time he called upon me was become much shorter than usual, and it was hurried on the least exertion.

exertion. For the preceding three weeks he had had night sweats to such a degree as to make his shirt wet; for the preceding fortnight he had experienced chills in the day-time. From the time at which the sweats had come upon him his sleep had been quite disturbed. He had lost flesh considerably, and was grown far weaker than common. His appetite had not failed him; but he had experienced a great and troublesom flatulence in the stomach, which sometimes incommoded him much while he was eating. The urine was high coloured; thick towards the bottom after standing. His tongue was moderately clean. Pulse at nine A. M. 98.

"He had a cough for some length of time the preceding winter; unaccompanied however, with pain in the chest, night sweats, or loss of flesh.

"I ordered eight grains of uva-ursi to be taken in warm milk, thrice in the day.

"December 15. Cough rather better. A pretty free expectoration with it; the expectoration mucus. Breath equally short. A little more pain about the right side of the chest. Less sense of chilliness. Perspirations not quite so great. Pulse 98, between nine and ten A. M. Tongue moderately clean. Bowels rather confined.

"He was directed to take fifteen grains of rhubarb and two of calomel, on the following morning. The uva-ursi was continued thrice in the day, in doses of ten grains each.

"Dec. 20. Cough a little improved since the 15th. Expectoration smaller in quantity. Breath not quite so short. Less pain about the chest; perspiration diminished; no chills; pulse at nine A. M. 88; tongue clean; bowels rather confined; still.

"Twelve grains of the uva-ursi were directed, thrice in the day; and a pill, consisting of four grains of the compound extract of coloquintida, every night.

"Dec. 26. Cough somewhat better; expectoration diminished; breath as on the 20th; pain in the chest less; he was now able to lie on the right side. The perspirations had been considerable one or two nights. No chills; strength improved; he remarked that from the gradual manner in which his strength had been declining when he first applied to me, he had reckoned upon being under the necessity of giving up work entirely, about this time; but that now he began to find work less fatiguing. He slept much better; flatulence in the stomach abated; urine less high coloured. Pulse 85, between nine and ten A. M. Tongue clean. The bowels having been rather open he had not taken the pill regularly.

"To take fifteen grains of uva-ursi thrice in the day; the compound extract of coloquintida occasionally.

"Jan. 7, 1805. The cough had grown gradually better till about January 3 when it was increased by long and imprudent exposure to the weather. It was now more troublesome than at the date of the last report. Expectoration small; the breath, notwithstanding, notwithstanding, was improved. No pain in the chest. He could sleep on either side, and he obtained twice as much sleep as when I first saw him. No perspirations; no chills; strength improving; less flatulence in the stomach; bowels regular without the pill; urine light coloured; pulse at ten A. M. 90; tongue clean.

"To take seventeen grains of the uva-ursi thrice in the day."

Under this course the phthisical symptoms continued to decline; and in observations on the case, Dr. Bourne expresses strong and apparently well grounded hopes of "confirmed recovery."

The four chapters immediately succeeding that containing the history of the cases above referred to, are composed of remarks on each particular affection, and of general observations on pulmonary and hectic disorders. These remarks display an extensive and intimate acquaintance with the characters of phthisical affections, and afford abundant evidences of correctness in judgment with respect to the general pathology of lymphatic and other diseases.

Dr. Bourne, in his observations on those cases which are not considered as instances of true pulmonary consumption, investigates the distinctive characters of pulmonic ulcer, which he divides into 1. Truly phthisical. 2. That consequent upon violent inflammation of the lungs; and, lastly, that which is almost imperceptibly generated, and which he terms *pulmonis vomica insidosa*. We regret that our limits prevent us from extracting our author's observations in detail, on the important subject of pulmonary ulceration.

The seventh chapter of the work is entitled Pharmaceutical and Practical Observations on the Uva-Ursi, in which a series of elaborate experiments are related, which were instituted for the purpose of ascertaining the respective powers of water and spirit in extracting the medicinal virtues of this plant. Dr. Bourne prefers the mode of administration in the form of powder, and adds, that in the cautious dose in which he has given it, "the fresh powder has in general agreed very well with the stomach." Should however the delicacy of this organ, or other causes, render this form of the medicine exceptionable, Dr. B. suggests the propriety of administering "the watery and spirituous preparations together, in order that patients may have the full advantage of both."

From what has been said on the general nature of the present work, our readers will infer, that we deem it worthy an attentive perusal, independantly of its relation to the medicinal agent which its principal object is to recommend. A character of extreme candour and great moderation pervades the pages of our author, which are commendable in proportion to its rarity in treatises published for the purpose of introducing a new remedy to public or professional notice. On uva ursi, in pulmonary affections of a consumptive nature, our experience does not authorise an opinion independently of that formed by a perusal of the work now before us. This plant appears however to be rather adapted to that form of phthisis, in which what are termed inflammatory symptoms are not not particularly conspicuous. On the occurrence of catarrhal irritation it was frequently found, necessary to interrupt the general course of treatment until the newly generated excitement had been considerably abated by the administration of other agents, and, as we have already observed, a combination of such medicines with the uva-ursi, was, in the majority of recorded cases, administered by Dr. Bourne.

---

*An Enquiry into the Nature and Action of Cancer, with a View to the establishing of a regular Mode of curing that Disease by natural Separation.* By SAMUEL YOUNG, Member of the Royal College of Surgeons, London; small octavo, pp. 130. London, 1805.

THE title of this performance gave us hopes that a regular practitioner had proposed something new in the treatment of an incurable disease. We shall leave Mr. Young, as far as is consistent with our limits, to speak for himself.

A Preface is supposed to speak the intentions of an author in the conduct of his work, it at least should show what he undertakes; and if he fulfils as much, the reader has no right to complain. The following is our author's PREFACE.

"On the nature of a disease hitherto so little determined as CANCER, *any* opinions should seem more or less important. It is possible that even error may, in some way, tend to aid the progress of inquiry; and since mere *supposition* has been tolerated, I trust that an attempt to exhibit the origin, progress, and nature of this disease, in some more distinguishable and relative form, will not be deemed *frivolous*.

"In this attempt, I have followed NATURE, as closely as the allowed difficulties of the subject would permit; and, however deficient the effort, I cannot but feel gratified if, out of such a chaos, I have selected a *beginning*, and an *end;* and have endeavoured to establish something like *connexion*, which more mature and accurate investigations may happily perfect.

"I offer this little work, therefore, rather as a figure on which the eye may rest, and on which the judgment, if not determined, may be safely exercised;—as the first rude sketch of a system, from which there may, one day, result some distinct and explicit criteria of this disease; as the faint penciling serves to direct the artist to particular form and subsequent elaboration.

"In drawing *my* outline, I have studiously avoided minutiæ; and while endeavouring to discover a principle for the better understanding, and towards a more regularly defined mode of treating Cancer, have refrained from any practical observations which might might prejudice the general question, by leading to partial disquisitions. The profile once struck, particular features may be more accurately filled up, and the minor touches hereafter be more effectively given.

"Be this tract considered then only as the *prospectus* of a zealous individual, on which every candid observation it may have the honour to attract will be gratefully adopted, or, at all events, respectfully considered."

A "General State of the Question" follows, in which, after remarking the language of writers on cancers, we are told that "such leading features as specific matter, constitutional action, transitive, hereditary natures cannot be admitted, particularly the two first, without general and determined *evidence*," which he conceives is wanting in this instance.

In taking a view of cancer as "a disease arising from specific virus and having a constitutional action," the author finds no difficulty in showing the fallacy of all the arguments brought to establish the infectious property of the discharge, and also the contamination of the constitution. Two very useful practical cases are given. These, as well as his other cases, are so well related and so pointedly introduced, that we much regret the author has not more frequently illustrated his subject in the same manner.

Chap. 2, contains an examination, "of facts which have been supposed to prove the specific quality of cancerous matter, and the constitutional nature of the disease." In this some playful remarks are made on the situation of Tulpius and of Mr. Smith, of St. Thomas's. The former conceived his throat affected by inhaling the effluvia of cancer, the latter by tasting the cancerous matter. One of Mr. Gooch's cases follows, with Mr. Pearson's remarks on the same. Mr. Gooch gave two other cases in which there could be no doubt that the application of cancerous virus had produced an ill-conditioned local ulcer. Though neither of these cases prove that cancer will produce cancer, yet they prove with a certainty that the pus may prove infectious, and consequently are more deserving notice than the long story about a child swallowing cancerous matter, and the full and particular account of her state of health for the rest of her life. This chapter concludes with some remarks on Dr. Hamilton's Observations on Scrofulous Affections, &c.

Chap. 3, "Of strumous glands, and further evidence against the specific virus of cancer. In this, another of Mr. Gooch's cases is brought forward. A quotation is given from a Dr. Nesbit, (whose treatise is said to contain nothing like an opinion) to prove that the disease of cancer does not rest on any specific virus. If a Dr. Nesbit's experiments on himself were worth transcribing, there was no occasion for his or Mr. Y's opinions, since those experiments are alone decisive of the question.

Chap. 4. "Of transitive, critical, and hereditary natures in cancer." Here our author remarks, that such is the contradictory accounts accounts of this disease, that each writer, for want of a path, seems to have wandered wherever his fancy led. Some instances of this are given from a few authors, and it is not difficult to show how unsatisfactory their reasoning proves. On the question concerning the hereditary nature, we meet with some confusion of terms, for want of an accurate distinction between "disposition" and predisposition.

The 5th chapter, containing "the Theory of Cancer," begins with showing what is now pretty generally admitted, that the disease depends not on any previous alteration in the juices, but in a change of action. "I would ask," says our author, "where is the disposition to hæpatized ammonia (see Dr. Crawford and others) to be found, when the origin of a cancer is laid in the breast of a girl of sixteen, by a blow." To this we would answer, that Dr. Crawford, to whose diligence and fidelity we are so much indebted for an accurate analysis, never considered that he had discovered any properties in cancerous matter, which are not to be met with in other foul ulcers; and as to the blow producing a disposition to the production of cancerous materials: all that the warmest advocates for the hereditary nature of cancer can insist on is—that such a blow would only produce cancer in a constitution predisposed to the disease. But we shall leave our author to give his own theory.

"Let us then suppose an injury sustained in such a part, not sufficient to stimulate *the common* mass into action, (so that inflammation and abscess might be the consequence,) yet so far effectual as to rouse one of these *little susceptible glands*:—Nineteen in twenty, perhaps, of such instances might happen without any ill consequences! or, at the worst, might proceed to a certain point, and then be resolved by absorption: yet a twentieth might become so entangled, that a *permanent* obstruction of one or two of these glands might follow; and here the mischief would commence. Though the œconomy of the general mass would not be deranged by such partial injury, yet still the obstructed gland must, in such a circumstance, be considered as a system in itself, endeavouring to regain its equilibrium; and to such struggles may be attributed the progress of the disease, by new actions being acquired.

"Thus it would proceed till its accumulation became a cause of irritation to the contiguous parts. Here a new field of action would take place. From such irritation the neighbouring glands would at length be driven into a similar complication of acquired actions, whilst the connecting cellular and adipose would undergo a more simple alteration of their structure by inflammatory obliteration and condensation. Things would thus go on, till the *internal pressure* became a *cause of irritation* to the *external covering*; and then an effort would be made, by the bursting of the integuments, to dislodge the whole offending mass.

"And here, undoubtedly, a *natural cure* would be effected, if the *diseased mass* were under the circumstances of *common exfoliation*: but the living principle not being destroyed in the mass, as in exfoliating bone, a separation could not be effected; and the various disjointed actions, now brought to the surface, would still pursue their course, presenting all the deformities of a cancerous sore."

The sixth chapter is on "parts more especially open to cancer," and further enquiries on the foregoing subjects. The parts most liable to cancer, our author conceives, are those which are most complicated, and principally, as he quotes from Dr. Baillie, parts the most glandular. In the remarks on that equally correct and perspicuous writer, it is very extraordinary that Mr. Young should still find so many difficulties in admitting, that where similar external causes do not produce the same effect on different people, we must look to some cause of difference in the people themselves.

"If cancer were a disease *suddenly* produced, then a predisposed state of parts might naturally be attributed; but we find it a disease resulting, more or less, from long and continued injuries, progressively altering natural structure. I agree cordially in the opinion, that a man's stomach is more predisposed to take on a cancerous action after ten years irregular dram-drinking, than it was in the first instance. I also agree (if the word is insisted upon) that a woman's breast, at a certain advanced age, is more "predisposed" to this disease than when she was much younger; or that the uterus of an aged woman is more open to cancerous affection than that of a young one; or that the prostate of an old debauchee is more liable to become schirrous than that of a young rake. But all this proves nothing with respect to the question of predisposition, as attaching that peculiarity to the cancerous action *itself*, which the author of the "Morbid Anatomy" would seem to infer."

How, we would ask, can such an inference be drawn from Dr. Baillie's words, as quoted by our author. "This affection of the stomach, says Dr. B. is not very uncommon towards an advanced period of life, and I think it more frequently met with in men than in women. This, perhaps, arises from the greater intemperance of one sex than the other. There must be added (says the Doctor), a considerable predisposition of the parts towards this disease."

"It cannot possibly be denied, answers our author, that a stomach may be *disposed* to take on the cancerous action before a glass of brandy ever entered it." But if brandy is the cause of this cancerous action, we do deny it; and if we understand Dr. Baillie, we conceive he would deny it also. When a cancerous *disposition* is formed, a cancerous action must follow, more or less slowly, according to the *predisposition* of the subject. If this is not intelligible, we would remark, that as brandy does not produce cancer in every stomach, it can only produce it in such as are *predisposed* to the disease. If habitual intemperance has so far altered the actions of a part as to prevent its resuming its proper functions, it must ultimately fall into that disease to which it is predisposed, and as such a cause (intemperance), does not produce similar effects in all, the effects must depend on some original structure or mode of action, action, which from our ignorance of both, we cannot better designate than by the t rm *predisposition.*

The same may be urged concerning the female breast, at a more advanced age, or after an injury. As these are not sufficient to produce cancer in all women, they can only do it in such as are predisposed to it. Even these causes, according to Mr. Young, do not immediately produce cancer, but only what he calls obstructions, and a series of actions or efforts, which end in cancer. As we are totally ignorant of the nature of these obstructions, as all that we know is, that a state of parts is produced, which is not at first cancer, or even scirrhous; we conceive the general term, disposition to cancer, may answer the purpose as well as any other. If this is the case, predisposition is the effect of original formation. Disposition, the immediate consequence of injury, or any other change in the part which ends in scirrhous or cancer.

We have said enough to give a sufficient insight into Mr. Young's Theory of Cancer; we shall conclude by remarking, that he stands upon as good grounds as many of his competitors. As to the performance itself, we must admit, it shows no inconsiderable industry, and so much acuteness, that we are encouraged to expect hereafter something more finished from the same quarter.

---

### *The Edinburgh Journal, No. IV.*

Article 1, is " *A Case of Hepatitis followed by bilious Expectoration.* By Alexander Monro, Jun. M. D. F. R. S. Professor of Anatomy and Surgery in the University of Edinburgh."

In this Case it appears, that pus formed in the liver was expectorated by the lungs. The arguments to prove the nature and source of the expectorated matter are sufficiently conclusive, though the fact could not be reduced to anatomical experiment, as the patient was alive when the account was written. He had recovered from three relapses, and was probably convalescent from the fourth, as he had left Edinburgh. Some judicious remarks are annexed, and an account of the chemical effect of different agents on the substance expectorated.

Article 2. " *Account of a peculiar Structure of the Membrane lining the Urethra, and of two supposed Hermaphrodites.* By John Barclay, M. D. Lecturer on Anatomy."

" Laying open the urethra of a man from the side of the corpora cavernosa penis, and observing the orifices of two lacunæ, which struck me as somewhat larger than ordinary, I applied the point of a blow-pipe to one, to see how far it would admit the air. On impelling the air with a slight degree of force, it entered the cells of the thin membrane lining the corpus spongiosum urethræ, and exhibited these cells as a kind of irregular vessels or canals. To preserve the appearance which had thus

 been been produced, I withdrew the blow-pipe and injected mercury, and observed distinctly, that though many of the cells communicated laterally, yet none of the injection flowed out through the cut margin of the urethral membrane, nor entered the cells of the corpus spongiosum, nor any of the venous or arterial vessels; that the cells, however, must have opened laterally into the cut edges of the membrane is evident, and the escape of the mercury through its lateral passages was in all probability prevented only by the retraction of the membrane itself. As the injection in this instance, however, furnished no proof of a communication, where certainly a communication existed, I was not authorised to deny a connexion between these cells and those of the corpus spongiosum urethræ, and more especially when I recollected that the communication between veins and arteries at their extremities cannot always be demonstrated, even by the minutest injections. The success of injections depends upon a variety of circumstances; and we proceed on a rash hypothesis when we imagine that our injections flow in the dead as freely and minutely as the different fluids in the living body. Immediately after the death of a dog, I have often injected the lymphatics of the cord from the spermatic artery, without any appearance of rupture that I could perceive; on plunging a dog into warm water as soon as he was killed, I have likewise succeeded in throwing even a coarse injection from the arteries into the veins; and have still a preparation of a Sapajous ape, killed and injected in similar circumstances, where all the veins of the sacral extremities, many of the pelvis, and the vena cava, to the place where it receives the emulgents, were completely filled from throwing the injection sacrad into a part of the abdominal aorta.

"In these instances, the event, were I to reason from experience, would have been different had I allowed the animals to cool, and tried the injection a day or two after.

"But although the injection in the case of the urethra neither flowed through the edges of the membrane nor into the cells of the corpus spongiosum, it flowed freely through the orifice of a lacuna, near the proximal extremity of the penis. Into this lacuna I likewise introduced the injecting pipe, but could not make the mercury flow with the same ease from the proximal to the distal as I had done from the distal to the proximal extremity; this seemed to imply, that in the communication between the cells there was something that performed an office resembling that of valves, though the difference of appearance, and the size of the lacunæ by which they opened at both extremities into the urethra, prevented me from concluding that they were lymphatics, in either a natural or diseased state.

"The preparation which I preserve is considerably disfigured, from drying, and from the escape of the mercury; but the drawing, which was done by Mr. Anthony Stewart, an ingenious artist, rep resents the appearance with great accuracy.

"I

"I have repeated the experiment on several other urethræ since, and have often failed; where I succeeded, I have found the canals considerably different in different individuals; and been led to conclude that this difference in structure, whether original or contracted by disease, has contributed not a little, with a difference of constitution, &c. to vary the nature of strictures and gonorrhœas, as well as the effects which result from the introduction of bougies."

The subjects of supposed cases of hermaphrodites were a pig and a lamb, in each of whom the urethra terminated in the perinæum.

Article 3. "*Case of a Bony Tumour, successfully extracted from the Orbit of the Eye.* By THOMAS LUCAS, Surgeon, at Stirling." This is an interesting little case, and is rendered the more useful from the following article:

Article 4. "*Analysis of the Bone extracted in the preceding Case.* By ANDREW DUNCAN, junr. M. D.

By this it appears, that the above substance perfectly resembled common bone in all its component parts, excepting, that the quantity of cartilage and phosphate of lime were greater in proportion to the carbonate of lime in the new formed substance than in the full formed bone of an adult subject.

Article 5. "*Drawings, with the Description of an Instrument for extracting Polypi from the Nose.* By WILLIAM ROBERTSON, one of the Surgeons to the Kelso Dispensary."

This instrument is well contrived, its plan does not materially differ from one which has been often used, excepting that harpsichord wire is substituted for string, and in our opinion with advantage.

Article 6. "*Case of a* Blue Girl, *with Dissection.* Communicated by ALEXANDER MARCET, M. D. one of the Physicians to Guy's Hospital."

We extract the Introduction to this Case, in order to show how little the practitioners in the London Hospitals are aware of the advantages they enjoy in the vast variety of new unrecorded cases with which they are perpetually meeting.

"During a course of Clinical lectures, at Guy's Hospital, last spring, I had the opportunity of noticing one of those singular instances in which the skin, all over the surface of the body, becomes tinged of a purple colour, in consequence of internal disease. The patient died, and the examination of the body offered some uncommon and unexpected appearances. One of the Editors of your Journal having heard of this case, expressed a wish that I should send you an account of it; and this I have now the pleasure of doing, more with a view to comply with his request, than from an idea that it will be found sufficiently interesting for your valuable publication."

The patient was about twenty-one years old, always subject to dyspnœa during the winter. The following is the Report after her admission into the house:

"The principal complaints which she now makes are, a wheezing cough, which occurs very frequently, but is not strong, apparently from her wanting strength to cough out; great shortness of breath, which is in some degree relieved when she sits up; very great prostration of strength; loss of appetite. She does not expectorate much, nor does she complain of pain in any part. Her hands and legs are œdematous. Her colour, in every part, is purple; but this is more peculiarly conspicuous in the hands, the lower part of the legs, the feet, and face. Her mother says, that the swelling and purple colour came on in one day, about seven weeks ago, and that she had previously complained of no peculiar symptoms. She is very thirsty. Bowels open. She generally dozes almost the whole day, but is very restless at night. She can lie best on her back, when her head and body are raised. She can, however, also lie well on the right side, but scarcely at all on the left. Her legs feel cold, both to herself and to those who touch them. The other parts of her body are of the usual warmth. Pulse 120, and appears regular; but it is extremely weak and small, and is hardly perceptible at the wrist. She has been under medical treatm nt, during the last seven weeks, without the least benefit, and has now a sore mouth, in consequence of mercurials which have been given.

"Immediately at her admission into the hospital, a blister was applied to the chest, and an attempt was made to promote the secretion of urine, and relieve her breathing, by various diuretics. The next day I made the following report:

"The colour is still more conspicuous than yesterday, and in the hands particularly; it is such as could scarcely be exceeded by any purple paint. It is, however, not near so intense on the trunk of the body. She lies on her back, with her head and body raised, and answers questions sensibly, though in a very faint voice; and any attempt to speak seems to be a very painful exertion to her. Her breathing is extremely quick, but feeble; and the contractions and dilatations of her chest are scarcely peceiveable. Her pulse is not to be felt at either wrist, and cannot be counted in any of the arteries. She says she is perfectly free from pain, and complains only of excessive shortness of breath. The blister has risen well, but she has had no passage in her bowels since yesterday, and has made but a small quantity of urine.

"She died a few hours after this report. Soon after death, the blue colour of the body gradually diminished, and the next day it had almost entirely disappeared.

"The

"The body having been opened, and the dissection carefully conducted by my collèague, Mr. Astley Cooper, we observed the following appearances, which were immediately recorded:

"ABDOMEN.—About half a pint of water was found in the cavity of the abdomen.

"The liver had been slightly inflamed, and had contracted morbid adhesions to the peritoneum.

"The vessels of the coats of the stomach and intestines were turgid with purple blood; but, in all other respects, the abdominal viscera were sound.

"THORAX.—The pericardium contained about an ounce and a half of water.

"The heart seemed somewhat enlarged, more completely filling the cavity of the pericardium than usual. In both sides it was turgid with blood of an extremely dark hue, and its coronary vessels were distended with blood of the same colour.

"*There was no unnatural communication whatever between the cavities of the heart, and its valves were all in a perfect and natural state.*

"*The lungs adhered every where to the inner surface of the chest, to the diaphragm, and to the pleura covering the pericardium.* These adhesions were some of them of long standing, others just forming; and these last were composed of large quantities of coagulable lymph, having the appearance and texture of jelly, and exhibiting signs of incipient organization.

"The lungs seemed distended with an unusual quantity of dark-coloured blood, but were free from tubercles, or any other appearance of disease: their substance seemed rather more compact than usual, particularly in the left lobe; but still it did not sink in water."

Some judicious remarks follow, in which Dr. Marcet admits, that the general expectation of the result from dissection, was very different to what actually occurred. This blue appearance could only arise from a want of oxygenation in the blood. The cause therefore must have been some impediment in the free circulation through the lungs. That organ is so much more considerable than what is absolutely necessary for the mere purposes of oxygenating the blood, that such a defect rarely occurs, if the heart is not prevented from its functions in transmitting the blood through the pulmonary vessels and the larger arteries. But in this instance, though the heart was perfect, yet the very close adhesion of the lungs to the inner surface of the chest prevented their expansion.

Our author makes some judicious remarks on finding the foramen ovale closed: to show that this blue appearance does not necessarily depend on the communication between the two sides of the heart remaining open. We have often suspected that where this opening has been detected, attended with an imperfect circulation, lation, it has been retained from necessity in consequence of some malformation which existed, and which, from the time that the lungs came into action, prevented the regular transmission of the blood through them.

Another remark we wish this ingenious author and his coadjutors to re-consider: He says,

"With regard to the purple colour gradually disappearing after death, it cannot, I think, be accounted for upon any other principle than from the muscular action of the arteries continuing for some time after death, and forcing the blood towards the larger branches, which, being under the more immediate influence of the heart, do not enjoy a muscular power proportional to that of the smaller ramifications."

The cause here assigned may be a sufficient one, because we cannot exactly ascertain before putrefaction commences, whether absolute universal death has taken place; nor till after stiffening and subsequent relaxation, whether muscular motion has ceased. But it is, at least, worth recollecting, that during life, the purple colour in the face, and even in the lips, is not changed in cases of temporary suffocation, unless the action of the lungs is restored: yet Dr. Priestly found that the air passing through the pores of a dead bladder was sufficient to give the crimson colour to the contained blood. Hence it may be doubtful, whether the blood in the extreme vessels may not, after death, become oxygenated by attracting gas through the pores of the skin.

Article 7. "*Case of Stricture in the Urethra removed by the Caustic Bougies, with an Account of the Appearance on Dissection 18 months afterwards.* By RICHARD CARMICHAEL, Surgeon to St. George's Dispensary, Dublin."

The most important part of this interesting paper is the account of the dissection. We shall also subjoin our author's practical remarks on the manner of applying the caustic.

"In presence of Dr. Toole, physician to St. George's Dispensary and Fever Hospital, who recollected his case, and other medical gentlemen, I accurately examined the urethra, but could trace no vestige of the strictures, except a little roughness where the second stricture had been situated, exactly behind the bulb. It was so trifling that, had we not been acquainted with his former complaints, we should have taken no notice of it. The lining membrane of the urethra had been perfectly restored.

"I shall make no comments on these satisfactory and unequivocal appearances of the urethra, which so forcibly indicate the superiority of the caustic over the common bougie. But the disrepute into which this mode of cure has fallen may be in a great measure accounted for, when we consider that, like every other powerful remedy in medicine and surgery, it is liable to be abused by a timid, unskilful, or ill-timed application. And, in the first place, I cannot approve of the timidity of the surgeon, who conceives it sufficient merely to touch the stricture, and instantly instantly withdraw the bougie. A little consideration convinces us that this undecided conduct occasions the nitrate of silver to act as a stimulus, and not as an escharotic; thus producing the very consequences most to be apprehended, inflammation, obstruction, and fistula.

"In the preceding case, and in every other which has since occurred to me, I applied the armed bougie steadily to the stricture *for four* or *five minutes*, and never experienced any inconvenience from that mode of application; for the surface of the caustic is necessarily so small, that if the period of the application were equally so, a sufficient quantity for the purpose intended would never be dissolved or brought to act upon the stricture.

"But too bold a use of this bougie is as much, if not more, to be avoided; and the endeavour to force it through the passage of the urethra, when it happens to be affected with spasmodic contractions, must produce incalculable mischief. On those contractions taking place, it should be instantly withdrawn, lest the caustic should dissolve on the sound part of the urethra, which is far more susceptible of inflammation than the diseased parts, those on dissection being found of a hard, ligamentous, or cartilaginous nature.

"Considerable discrimination will be necessary in selecting a proper time for applying the caustic; as it should be avoided when pain, inflammation, or ulceration, encompass the diseased parts, and obstruct the urine, while matter mixed with blood runs from the urethra. These complaints are first to be removed by warm fomentations, and other remedies of a similar nature, before bougies of any kind are to be used, but particularly the caustic; for, if employed during such a state of excitement, we need not be surprised that such consequences should follow as violent inflammation, mortification, and dangerous or fatal hæmorrhagies.

"Without meaning to enter into any controversy, or to cast any reflection on those gentlemen with whom I may differ on the subject, I beg leave to express my thorough conviction, that the caustic bougie will be found, on a skilful and impartial trial, a safe and efficient remedy, where every other has been in vain persisted in for years; and although the older surgeons endeavoured to apply caustic to strictures, they were much better acquainted with its virtues, than with the means of bringing them into action; and I can scarcely express how much I admire Mr. Home's elegant invention of the conveyance of this powerful escharotic to the affected part, with which, simple as it may seem, the world has to regret it was not sooner acquainted; but which, if humility like mine may venture to predict it, will be tried wherever the principles of its action are known, and efficacious wherever it is tried."

Article 8. "*Observations on the Cure of those unnatural Articulations*

*ticulations which are sometimes the Consequence of Fractures in the Extremities, with Cases.* By ANDREW INGLIS, M. D. one of the Surgeons to the Royal Infirmary of Edinburgh."

Where the form of the new articulation will admit of it, the author proposes rubbing the two ends of the bone together, in order to produce inflammation and consequent union. In other cases, where the distortion of the leg required it, pressure was used with equal success. Where these means are prevented by the bones overlapping each other, Dr. Inglis has succeeded in the operation proposed, first by Mr. White, and since frequently adopted. On this occasion we have a quotation from Cit. Boyer, who considers the operation as impracticable in the leg and fore arm. In this it appears that Cit. Boyer was mistaken, but he has afforded some other useful hints, which it would have been candid in the writer to have acknowledged. Dr. Inglis had an opportunity of dissecting a leg after the operation had been successfully employed, and found the two bones consolidated.

These are all that can strictly be called the original Communications. An Essay is subjoined by the Enquirer, "Is there any certainty in Medical Science?"—We are sorry to remark so many typographical errors in this Number.

